# Appointment of Dr. Samuel Jackson, Late of Northumberland

**Published:** 1850-11

**Authors:** 


DR. SAMUEL JACKSON, late of Northumberland, has been appointed one of the physicians to St. Joseph's Hospital, in place of Professor Jackson, resigned.

---

DR. ALFRED STILLÉ of Philadelphia, has gone to Europe in consequence of impaired health. He carries with him the sincere wishes of many friends for his speedy restoration.

